# Functionally Competent, PD-1^+^ CD8^+^ Trm Cells Populate the Brain Following Local Antigen Encounter

**DOI:** 10.3389/fimmu.2020.595707

**Published:** 2021-02-02

**Authors:** Amalie Skak Schøller, Loulieta Nazerai, Jan Pravsgaard Christensen, Allan Randrup Thomsen

**Affiliations:** Department of Immunology and Microbiology, University of Copenhagen, Copenhagen, Denmark

**Keywords:** immune surveillance, CNS, tissue resident memory T cells, PD-1, adenovectors, viral infection

## Abstract

Expression of programmed cell death-1 receptor (PD-1) has traditionally been linked to T-cell exhaustion, as signaling *via* PD-1 dampens the functionality of T-cells upon repetitive antigen exposures during chronic infections. However, resent findings pointing to the involvement of PD-1 both in T-cell survival and in restraining immunopathology, challenge the concept of PD-1 solely as marker for T-cell exhaustion. Tissue resident memory T cells (Trms) hold unique effector qualities, but within a delicate organ like the CNS, these protective abilities could potentially be harmful. In contrast to their counterparts in many other tissues, brain derived CD8^+^ Trms have been found to uniformly and chronically express PD-1. In this study we utilized a recently established model system for generating CNS Trms in order to improve our understanding regarding the role of PD-1 expression by Trms inside the CNS. By intracerebral (i.c.) inoculation with a non-replicating adeno-viral vector, we induced a PD-1^hi^ CD8^+^ T cell memory population within the CNS. We found that PD-1 expression lowered the severity of clinical disease associated with the i.c. inoculation. Furthermore, high levels of PD-L1 expression were found on the infiltrating monocytes and macrophages as well as on the resident microglia, oligodendrocytes and astrocytes during the acute phase of the response. Additionally, we showed that the intensity of PD-1 expression correlates with local antigen encounter and found that PD-1 expression was associated with decreased CD8^+^ T cell memory formation in the CNS despite an increased number of infiltrating CD8^+^ T cells. Most importantly, our experiments revealed that despite expression of PD-1 and several additional markers linked to T-cell exhaustion, Tim-3, Lag-3 and CD39, the cells did not show signs of limited effector capacity. Collectively, these results endorse the increasing amount of evidence pointing to an immune-modifying role for PD-1 expression within the CNS, a mechanism we found to correlate with local antigen exposure.

## Introduction

The CNS might be the target of acute as well as persistent viral infections. Despite the immune restriction of the CNS, such infections along with certain autoimmune diseases, e.g., multiple sclerosis (MS), represent unique challenges for effective pathogen and immune control. Following resolution of a local CNS infection a first line of protective immunity against recurrent or reactivated brain-infections is provided by a subset of local memory cells termed tissue-resident memory T cells (Trms) (1). It is widely known that during the course of CNS inflammation, the number of CNS infiltrating leukocytes dramatically increases (2, 3). This is the case during acute infections, but also a consequence of Trm re-activation e.g. upon a flare-up of a latent viral CNS infection (4–6). Therefore, despite the critical importance of generating immunological memory within the CNS, the unique qualities that Trms possess needs to be tightly controlled to limit immunopathology. Programmed cell death-1 receptor (PD-1) is known as a marker for T-cell exhaustion (7, 8). PD-1 expression has particularly been studied in models of chronic viral infections such as that following infection with the highly invasive clone 13 of lymphocytic choriomeningitis virus (LCMV), which is associated with a high and prolonged antigenic load (6, 8–11). CD8^+^ T-cell exhaustion refers to a transformation of antigen-primed T-cells caused by repetitive TCR stimulation, leading to reduced effector capacity, including an impaired ability to produce pro-inflammatory cytokines such as IFN-γ and TNF-α (12). Exhausted T cells additionally upregulate a range of markers for terminal differentiation as well as inhibitory receptors, the expression of which has been found to correlate with T-cell dysfunction. These include: CD39, 2B4, Tim-3, Lag-3, PD-1 as well as increased expression of Eomes (Eomesodermin) (7, 13–19). PD-L1 and PD-L2 are the ligands for PD-1 (20, 21). Engagement of PD-1 with these ligands initiates the recruitment of phosphatases, which inactivate kinase-cascades and ultimately inhibit the pathways required for effector T-cell function (22, 23). While PD-L2 is only expressed on antigen presenting cells, accumulating evidence has shown that PD-L1 is also expressed on a number of other cell types including astrocytes and glial cells during CNS infection (24, 25). Although microglia cells traditionally have been thought of as pro-inflammatory, their expression of PD-L1 have been shown to dampen immune-mediated tissue damage in studies on viral encephalitis and experimental autoimmune encephalomyelitis (EAE) (24–28). These findings challenges the use of PD-1 solely as a marker for T-cell exhaustion and indicates that initiating the PD-1:PD-L1 pathway could be a mechanism implemented to limit inadvertent effector T-cell functions to avoid tissue-damage in this non-regenerative organ (22, 24, 28–30). The PD-1 promotor (*Pdcd-1*) has been found to undergo epigenetic changes at the methylation level during the different developmental stages of CD8^+^ T cells (31–33). A recent study by Wherry et al. demonstrated a disease specific relation for these epigenetic changes responsible for PD-1 expression (34). Accordingly, studies of chronic LCMV clone 13 infection revealed that upon resolution of the infection, the *Pdcd-1* promoter remained un-methylated, whereas upon resolution of an acute infection with LCMV Armstrong, the *Pdcd-1* promotor was re-methylated causing downregulation of PD-1 (32, 33, 35). Based on these epigenetic studies, PD-1 involvement in the Trm differentiation program has been suggested (27, 36). In this study, we utilized our previously established model system for inducing CD8^+^ T cell memory within the CNS (37). By intracranial inoculation (i.c.) with a non-replicating adeno-viral vector, we generated an immune response that mimics the response towards a non-lethal viral infection of the CNS. Due to the prolonged antigen exposure linked to the adenoviral infection (38, 39) we reasoned that this model could also serve as a model for certain CNS-directed autoimmune responses. In these settings, we asked whether PD-1 expression by brain localized CD8^+^ T cells correlates with T-cell intrinsic exhaustion or primarily operates *in situ* to restrain inadvertent effector T cell functions that might lead to cell damage inside this delicate organ. Furthermore, we aimed at enlightening the mechanisms contributing to PD-1 expression. Our results suggest a prominent role of local antigen encounter for initial PD-1 upregulation by the CD8^+^ T cells. Additionally, we show that PD-1 expression affected the severity of clinical disease associated with the i.c. inoculation as well as the memory potential of the CD8^+^ T cells. Most interestingly, our experiments revealed that despite their seemingly exhausted phenotype, memory CD8^+^ T cells maintained in the CNS did not show signs of T-cell intrinsic limitations in effector capacity.

## Materials and Methods

### Mice

All mice used in this study were between 6–12 weeks old when entering the experiments. Mice were housed under controlled (specific pathogen free) conditions in individually ventilated cages in an ALAAC accredited animal facility at the Panum Institute (Copenhagen, Denmark). Wild -type (WT) female C57BL/6 (C57BL/6JBomTac, H-2^b^) mice were purchased from Taconic Farms (Ry, Denmark), and PD-1 KO mice (B6.Cg-Pdcd1tm1.1Shr/J) were obtained from the Jackson laboratories (Bar Harbour, MA, USA). Mice from outside sources were always rested for at least 1 week before entering an experiment. All procedures were approved by the national animal ethics committee (The Animal Experiment Inspectorate) and were conducted in accordance with national Danish guidelines.

### Recombinant Adenoviral Vectors

A replication deficient adenoviral vector encoding the glycoprotein (GP) of lymphocytic choriomeningitis virus (LCMV) (AdIi-GP) was used to induce a potent CD8^+^ T cell response. This human serotype 5 recombinant adenoviral (Ad5) vector with an E1-deleted and E3 inactivated region was constructed and purified as described in (40, 41).

### Immunization and Challenge With Live Virus (LCMV)

Mice were immunized by i.c. inoculation of 2x10^7^ pfu/30 µL. For i.c. challenge of immunized mice, a volume 30 µL containing a dose of 10^3^ pfu LCMV Armstrong 53B was used; this dose is invariably lethal to unimmunized mice.

### *In Vivo* Depletion of CD8^+^ T Cells

When mice were depleted from CD8^+^ T cells, 200 µg/300 µL α-CD8 mAb (YTS169.4) where administered i.p. one day prior to AdIi-GP i.c. inoculaton (day -1). At day one (+1) and four (+4) after AdIi-GP i.c. inoculation, half the original dose α-CD8 mAb (YTS169.4) (100 µg/300 µL) were administered i.p. α-CD8 mAb (YTS169.4) were obtained from BioXcell.

### Blocking of T Cell Exit From the Lymph Nodes With FTY720

FTY720 was obtained from Sigma-Aldrich, and dissolved in water. For treatment, mice were allowed free access to drinking water containing FTY720 at a dose of 2.5 µg/mL; treatment intervals varied and are described in the experiments.

### *In Vivo* 5-Ethynyl-2’-Deoxyuridine (EdU)-Labeling

95% EdU were obtained from Sigma-Aldrich, and dissolved in water. Mice were given EdU in their drinking water at a dose of 22.5 µg/mL for varying periods of time as described in the experiments.

### Single Cell Preparation

Brains were aseptically removed after intracardial perfusion with 20mL PBS. Mice were deeply anaesthetized during this process *via* intraperitoneal (i.p.) injection of avertin (2,2,2 tribromoethanol in 2-methyl-2-butanol, 250mg/kg). Following the perfusion, brains were transferred to RPMI 1640 medium [supplemented with 1% L-glutamin, 1% penicillin, 1% streptomycin, 1% 2-mercaptoethanol (2-ME) and 10% fetal calf serum (FCS)]. To obtain single cell suspension brains were pressed through a 70-µm nylon cell strainer. Single cell suspension was then centrifuged at 400 g for 10 min at 4°C. The remaining pellet was vortexed and lymphocytes were separated on a 37% Percoll gradient during 20 min centrifugation at 2800 g, 20°C. The supernatant was then carefully aspired and the remaining pellet was vortexed and washed twice in RPMI 1640. An additional filtration through a 70-µm cell strainer was performed and finally the cells were resuspended in FACS medium (PBS, 1% BSA, 0.1% N_a_N_3_).

Spleens were aseptically removed and transferred to Hanks Balanced Salt Solution (HBSS). Single-cell suspensions were obtained by pressing the spleens through a 70 µm nylon cell strainer, followed by centrifugation and two washes in HBSS.

### Flow Cytometry Analysis

Approximately 2-5x10^6^ cells from the brain samples or 2x10^6^ splenocytes were transferred to wells of U-bottomed 96-well microtiter plates. For tetramer staining the cells were incubated for 20 min (RT, in the dark) with 50 μl FACS medium (PBS, 1% BSA, 0.1% N_a_N_3_) containing 10 µL relevant fluorochome labeled tetramers; these were kindly provided by S Buus and A Stryhn (this institute). To prevent unspecific binding cells were blocked with α-CD16/32 and subsequently incubated for 20 min (4°C, in the dark) with 50 µL brilliant violet staining buffer (Biolegend) containing conjugated antibodies (1:100) for the relevant cell-surface markers. Cells to be stained for intracellular granzyme B, T-bet or Eomes expression were permeabilized and stained according to the FoxP3 staining protocol from BD Biosciences. When analyzing the EdU incorporation cells were permeabilized and stained according to the Click-It™ Plus EdU Alexa Flour™ 647 Flow Cytometry Assay Kit from Invitrogen by Thermo Fisher Scientific. Cells were subsequently washed twice in wash media (PBS with 0.1% N_a_N_3_), resuspended in PBS and stored at 4°C until analysis. The general gating strategy regarding CNS derived cells is depicted in **Supplementary Figure 1**.

### Intracellular Cytokine Staining

When evaluating intracellular cytokine production, cells were first incubated for 5 hours (37 °C, 5% CO2) in 200 µL cell culture medium containing 50 IU/mL of IL-2 and 3 μM monensin in the presence of 1 μg/mL of the relevant peptides, LCMV GP_33-41_ or GP_276-286_. As a control, samples without peptide stimulation were included. Following incubation, cells were centrifuged (2000 rpm, 3 min), and washed in FACS buffer containing monensin (PBS containing 1% BSA, 0.1% NaN3 and 3 μM monensin). Then the cells were incubated for 20 min. (4 °C, dark) with 50 μl FACS/Monensin medium containing the relevant antibodies for surface staining (final dilution 1:100). Next, the cells were washed twice with PBS/monensin medium (3 μM monensin in PBS) and fixed in 200 μL 2% paraformaldehyde (PFA) for 15 min. (4 °C, dark). Subsequently, cells were washed with FACS/monensin medium and incubated for 10 min. (20 °C, dark) with 200 μl Saponin medium (PBS containing 0,5% Saponin). Subsequently, cells were incubated for 20 min. (4 °C, dark) in 50 μL Saponin medium containing the relevant intracellular antibodies (1:100), and then washed twice with Saponin medium. Finally, the cells were resuspended in PBS and stored at 4 °C until flow cytrometry analysis was performed.

The samples were analyzed using a FACS—Fortessa 3—or 5 cytometer (BD Biosciences) and subsequently gated and analyzed in FlowJo software version 10 (TreeStar).

### Reagents for Flow Cytometry

The following fluorochrome-conjugated Abs, purchased from Biolegend, eBiosciences or BD Bioscience as anti-mouse antibodies, were used for cell surface staining: α-CD8, α-CD11b, α-CD45.2, α-CD103, α-CD69, α-PD-1, α-PD-L1, α-CD127, α-CD43, α-CD39, α-Tim-3, α-Lag-3, α-CD244.2 (2B4), α-O4, α-ASCA2, and α-KLRG1. When staining for intracellular markers, the following fluorochrome-conjugated monoclonal antibodies were used: α-IFN-γ, α-IL-2, α-TNF-α, granzyme B, T-bet or Eomes. Incorporation of EdU was evaluated using the EdU Alexa Flour™ 647 antibody Click-It^™^ from Invitrogen by Thermo Fisher Scientific.

### Statistical Evaluation

Analyzes was performed using GraphPad Prism software (version 7). Quantitative results were compared using a nonparametric Mann-Whitney *U*-test; a p value of <0.05 was considered evidence of a statistically significant difference and was visualized by an asterix (*).

## Results

### CD8^+^ Trm Cells in the CNS Undergo Local Homeostatic Proliferation

In a previous study we showed that i.c. inoculation of a non-replicating adenoviral vector expressing a non-self antigen induced a stable population of antigen-specific CD8^+^ Trm cells inside the CNS (37). However, although these cells seemed to undergo prolonged proliferation based on expression of Ki-67, we wanted to confirm active cell division using a more direct technique and also to determine whether there was ongoing local proliferation.

**Figure 1:**
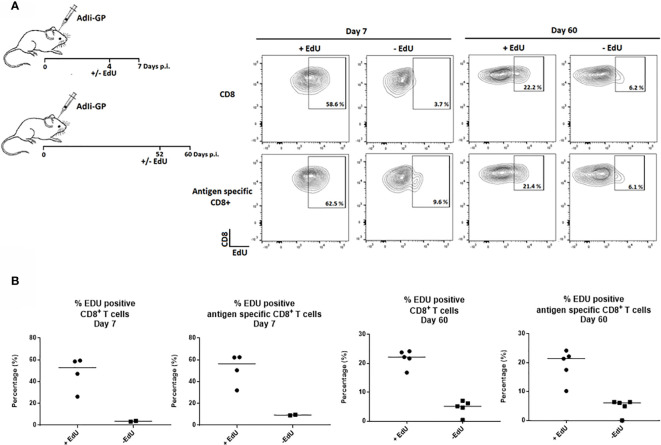
CD8^+^ Trm cells in the CNS include a subset of proliferating cells. Two groups of C57BL/6 mice were inoculated with AdIi-GP i.c. and half of the mice were administered EdU in their drinking water as indicated above. Brains were harvested at day 7 and 60 post i.c. **(A)** Representative plots of 2–5 mice/group at each time point, showing the percentage of CD8^+^—and antigen specific (tetramer^+^) CD8^+^ T cells positive for EdU. The mice without EdU administration were shown as control for background. **(B)** Percentages of EdU positive cells in individual mice, results are representative of 1 (day 7) or 2 (day 60) experiments.

Therefore, to study how the Trm population is sustained long-term, the proliferative activity of the CD8^+^ T cell population in the CNS was analyzed by an *in vivo* EdU-incorporation assay. Two groups of C57BL/6 mice were inoculated intracerebrally (i.c.) with 2x10^7^ pfu AdIi-GP, and brains were harvested at 7 and 60 days post inoculation (p.i.). Half of the mice were administered EdU in their drinking water for four or eight days, respectively, prior to cell extraction. The CD8^+^ - and antigen specific CD8^+^ T cells extracted from the CNS showed clear EdU incorporation, particularly during the effector-phase of the response (day 7 p.i.), but significant EdU incorporation was also found during the memory-phase (day 60 p.i.) (**Figure 1**).

**Figure 2:**
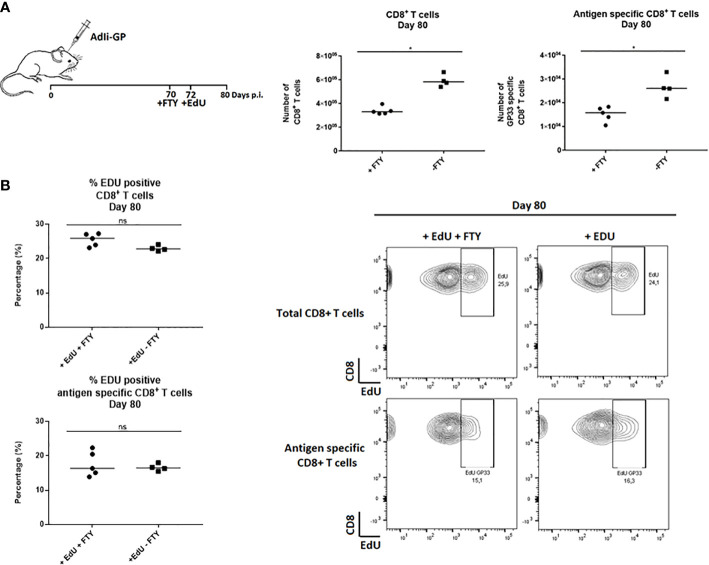
CD8^+^ Trm cells in the CNS undergo local homeostatic proliferation. Two groups of mice were inoculated i.c. with AdIi-GP and 10 days prior to brain extraction half of the mice were administered FTY720 in their drinking water. Eight days before brain extraction, all mice were administered EdU in their drinking water or in the FTY720/drinking water mixture, respectively. **(A)** The number of CNS infiltrating CD8^+^ T cells and antigen specific (tetramer^+^) CD8^+^ T cells were measured for both groups. Each dot represents an individual. Bars represent median. P>0.05 illustrated by * **(B)** The percentage of EdU positive CD8^+^ and antigen specific CD8^+^ T cells were analyzed day 80 post i.c. to compare EdU incorporation in the presence of FTY720 or when recruitments were allowed. Percentages and representative plots of 4–5/group are shown. Results are representative of 2 experiments.

To investigate whether the observed EdU incorporation during the memory phase reflected local CD8^+^ T-cell proliferation and not ongoing replenishment by circulating T cells recently derived from cells proliferating in the secondary lymphoid organs, FTY720 was added during the period of EdU incorporation. FTY720 has previously been found to inhibit the recruitment of activated CD8^+^ T cells to the site of antigen challenge (36, 42, 43). Two groups of mice were inoculated i.c. with AdIi-GP and 10 days prior to cell extraction half of the mice were administered FTY720 in their drinking water. Eight days pre cell extraction, all mice were administered EdU in their drinking water or in the FTY720/drinking water mixture. The number of CD8^+^ T cells and antigen specific CD8^+^ T cells were determined for both groups. Slightly fewer CD8^+^ T cells were recovered from the brain after FTY720 treatment (**Figure 2A**). However, despite inhibition of T-cell recruitment, the percentage of EdU incorporating CD8^+^ T cells extracted from the CNS was similar between the two groups (**Figure 2B**), indicating that the sustained proliferative ability of the CD8^+^ T cells in CNS did not simply reflect the arrival of cells that have recently divided in lymphoid organs. These results indicate that the CNS memory CD8^+^ T cell population retains the capacity to undergo local homeostatic proliferation.

### CD8^+^ Trm Cells From CNS Express a Range of Inhibitory Receptors Correlating With Signs of Recent Activation

**Figure 3:**
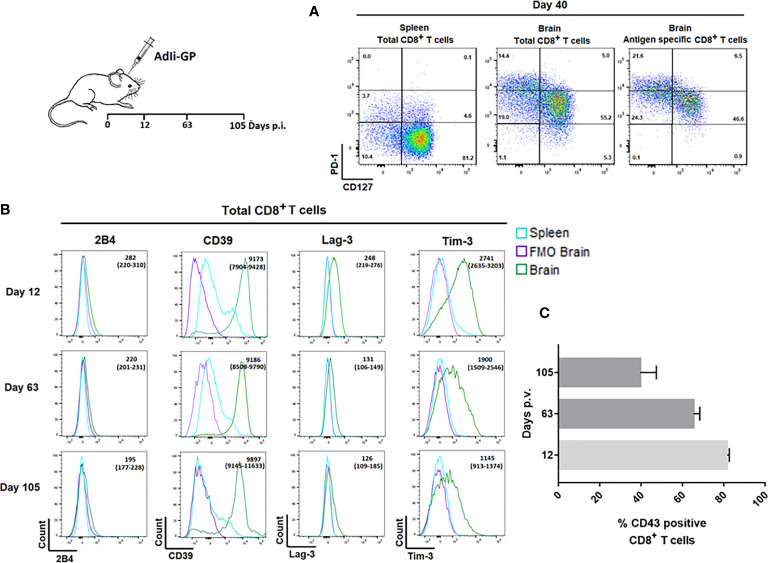
Expression of inhibitory receptors on CD8^+^ Trm cells correlate with signs of recent activation. Wild type C57BL/6 mice were inoculated with AdIi-GP i.c. **(A)** Representative plots of showing PD-1 and CD127 expression by CD8^+^ splenocytes, total CD8^+^ and the antigen specific (tetramer^+^) CD8^+^ T cells from CNS. Splenocytes are included for comparison. Results are representative of analyses of groups of mice carried out between 12 and 103 days after inoculation. **(B)** Representative histograms of 5 mice/group showing the expression of the inhibitory receptors 2B4, CD39, Lag-3 and Tim-3 by CNS infiltrating CD8^+^ T cells at twelve, 63 and 105 days post i.c. FMOs are included as controls for background. Medians of mean fluorescence intensities and ranges of expression of each marker for groups of 4-5 mice are depicted. Results are representative of 2-3 experiments for each time point. **(C)** Percentage of CD43^high^ CD8^+^ T cells as a function of time after virus inoculation. Columns represent medians +/− ranges of 4–5 mice.

Pertaining to the persistence of CD8^+^ memory T cells in the CNS, we have found that at least half of the CNS infiltrating CD8^+^ T cells display a so called memory precursor phenotype with expression of CD127, but not KLRG1 (1, 37). However, the expression of CD127 on part of the CD8^+^ T cells inside the CNS was reduced compared to the expression on their splenic counterparts (37). In view of reports suggesting that Trms isolated from the brains of mice with certain chronic neuroinfections express elevated levels of PD-1 (28, 33), we decided to investigate whether the reduced level of CD127 expression could be linked to PD-1 expression, and whether PD-1 expression could be essential in modulating the local response and memory generation. Mice were inoculated with AdIi-GP i.c., and brains and spleens were extracted on day 40. p.i. The expression of CD127 and PD-1 was analyzed for CD8^+^ T cells in the brain and spleen and for the antigen specific CD8^+^ T cells recruited to the CNS. Interestingly, CD8^+^ T cells isolated from the brain showed a markedly elevated expression of PD-1 compared to their counterparts in the spleen. Furthermore, cells with intermediate expression of PD-1 tended to express higher levels of CD127 (**Figure 3A**), this pattern was maintained over time (data not shown). Recent studies have suggested that Trms may express other phenotypic markers normally associated with exhaustion (33, 44–46). In order to carry out an in-depth analysis of the CNS localized CD8^+^ T cells with regard to markers of functional dysfunction, mice were inoculated with AdIi-GP i.c. On day 12, 63 and 105 p.i., brains and spleens were extracted, and surface expression of 2B4, CD39, Lag-3 and Tim-3 for the total number of CD8^+^ T cells were analyzed. Unlike 2B4, which we did not find to be expressed at any time-point, we observed that the CD8^+^ T cells from CNS also expressed CD39, as well as Lag-3 and Tim-3 (**Figure 3B**). Expression of Lag-3 and Tim-3 was evident throughout the observation period, although we noted a trend toward decreasing expression with time (**Figure 3B**). In contrast, CD39 expression did not decline markedly with time (**Figure 3B**).

We reasoned that the observed decline in Lag-3 and Tim-3 expression might reflect a decrease in the state of activation during the analyzed time-period. Consequently, we decided to follow the percentage of CD8^+^ T cells expressing CD43, as CD43 has been associated with recent CD8^+^ T-cell activation and proliferation (47, 48). For that reason, mice were inoculated with AdIi-GP i.c. and day 12, 63 and 105 p.i. respectively, brains were extracted and the expression of CD43 was analyzed. In agreement with our assumption, we found a decrease in the percentage of CD43 expressing CD8^+^ T cells from around 80% on day 12 to about 40% day 105 p.i. (**Figure 3C**).

### Recruited Immune—and Local Parenchymal Cells Express Increases Levels of PD-L1 During Acute CNS Inflammation but Declines Over Time

**Figure 4:**
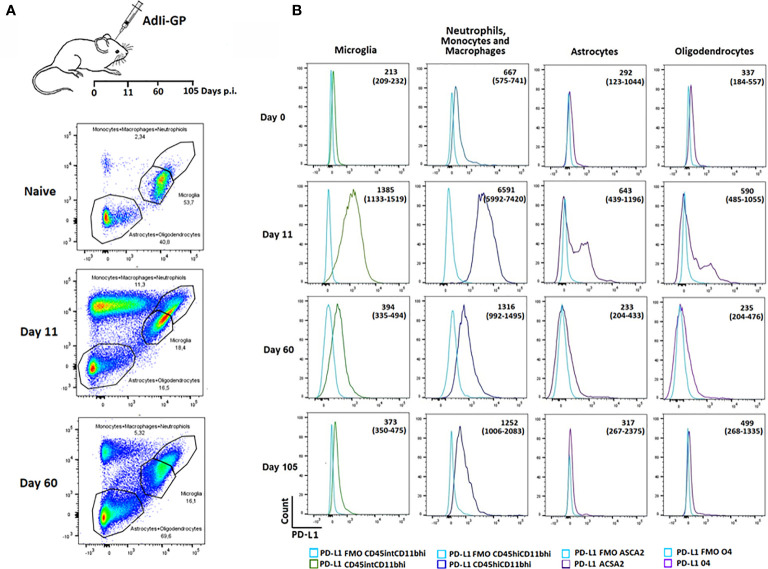
Recruited immune and local CNS cells express PD-L1 during acute CNS inflammation, but expression declines over time. Following inoculation with AdIi-GP i.c, infiltrating neutrophils, monocytes and macrophages as well microglial cells were categorized based on the expression of CD45.2 and CD11b. The CD45^low^CD11b^low^ cells were further subdivided into 04^+^ oligodendrocytes and ASCA2^+^ astrocytes. **(A)** Representative plots of 4-5 mice at each time point, showing the gating of the three major cell-subsets for naïve mice and mice taken at two time-points post inoculation. For representative plot showing the subdivision into olidendrocytes and astrocytes, please see **Supplementary Figure 2**. **(B)** Representative histogram of PD-L1 expression by the above mentioned cell subsets at day 0 (naïve mice), 11, 60 and 105, respectively. FMOs are shown in all histograms to indicate background. Medians of mean fluorescence intensities and ranges of PD-L1 expression for groups of 4-5 mice are included.

We next wanted to investigate whether PD-1 expression could affect the functionality of the T cells inside the CNS. PD-1 mediated inhibition would only work, if relevant ligands were expressed on cells potentially interacting with the CD8^+^ T cells. Consequently, we wanted to investigate whether local CNS – and/or infiltrating cells expressed PD-L1. Mice were inoculated i.c with AdIi-GP and brains were harvested on day 11 and 60 p.i. Based on the expression of the adhesion molecule CD11b, the leukocyte marker CD45 as well as ACSA-2 and O4, markers for astrocytes and oligodendrocytes, respectively (49, 50), microglia, astrocytes and oligodendrocytes as well as the infiltrating neutrophils/monocytes/macrophages were gated (**Figure 4A**). These subsets were then analyzed for expression of PD-L1. Interestingly, during the effector-phase of the T-cell response, all of the analyzed cell types expressed PD-L1 at an increased level compared to control animals (**Figure 4B**). It should also be noted that PD-L1 expression by local and recruited innate immune cells was markedly higher than what was found for astrocytes and oligodendrocytes, where only a portion of the total population expressed PD-L1 (**Figure 4B**). At 60 days p.i. PD-L1 expression was noted mainly on infiltrating monocytes/macrophages, and at 105 days p.i. expression on all cell population seemed to be further normalized (**Figure 4B**). These data supported a possible role for PD-L1:PD-1 interaction in regulating the induced, acute inflammatory activity inside the CNS.

### Local Antigen Encounter Increases PD-1 Expression by CD8^+^ Trm Cells in CNS

**Figure 5:**
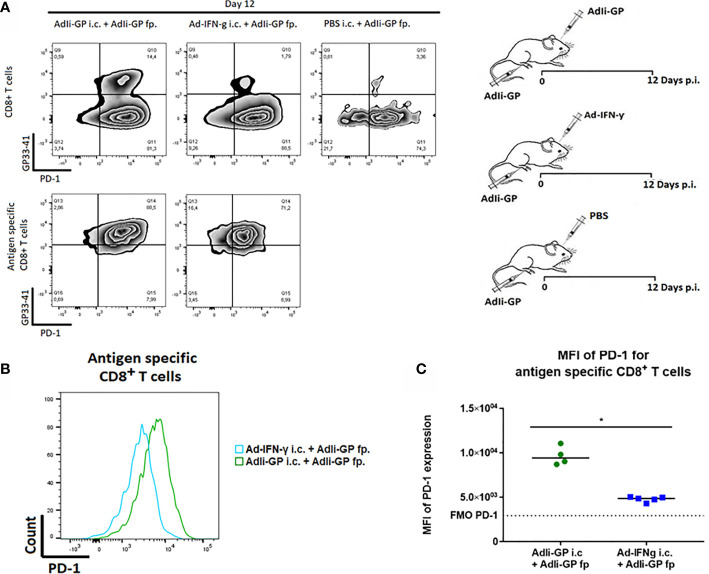
Local antigen encounter increases PD-1 expression on CD8^+^ Trm cells. Three groups of C57BL/6 mice were inoculated with AdIi-GP in the footpad (f.p.), combined with i.c. inoculation of either PBS for control, the same construct or an adeno-vector construct encoding the gene for murine IFN-γ expression. Day 12 post i.c. inoculation, brains were extracted and the expression of PD-1 was analyzed. **(A)** Representative plots of 5 mice showing PD-1 expression on total CD8^+^ T cells and antigen specific (tetramer^+^) CD8^+^ T cells respectively. Cut-offs are based on PD-1 FMOs for background expression in the brain. **(B)** Representative histograms of 5 mice illustrating PD-1 expression on antigen specific CD8^+^ T cells harvested from the brains of AdIi-GP f.p. vaccinated mice combined with ether AdIi-GP or Ad-IFN- γ i.c. inoculation. **(C)** Mean fluorescent intensity (MFI) for PD-1 expression on antigen specific CD8^+^ T cells isolated from brains of AdIi-GP f.p. vaccinated mice combined with ether AdIi-GP or Ad-IFN- γ i.c. inoculation. Background reflects MFI measured in PD-1 FMO from the brain. Each dot represents an individual, bars represents median. P>0.05 illustrated by *. Results are representative of 2 experiments.

Next we wanted to investigate the association between local antigen encounter versus bystander inflammation as key factors for inducing PD-1 expression by CNS localized CD8^+^ T cells. Consequently, three groups of C57BL/6 mice were inoculated with AdIi-GP in the footpad (f.p.). This inoculation was combined with an i.c. inoculation of either 30 µL PBS for control, the same construct or an adeno-vector construct encoding the gene for murine IFN-y, which cause the infected cells to produce this pro-inflammatory cytokine (51). Brains and spleens were harvested day 12 p.i. The expression of PD-1 on antigen specific CD8^+^ T cells was analyzed. In accordance with our previous findings, i.c. inoculation with the antigen expressing adenoviral-vector resulted in the establishment of a robust antigen-specific CD8^+^ T cell population in the CNS (37), which was dominated by cells expressing high levels of PD-1 (**Figure 5A**). However, when we analyzed the more limited number of antigen specific CD8^+^ T cells recruited non-specifically by IFN-γ, expression of PD-1 was also up-regulated, albeit to a lesser degree (**Figures 5A, B**). Thus, the MFI value for PD-1 expression was significantly higher when antigen was presented locally compared to the situation where only unspecific inflammation occurred (**Figure 5C**).

### PD-1 Modifies the T Cell–Mediated Immunopathology Caused by Intracerebral Inoculation of AdIi-GP

**Figure 6:**
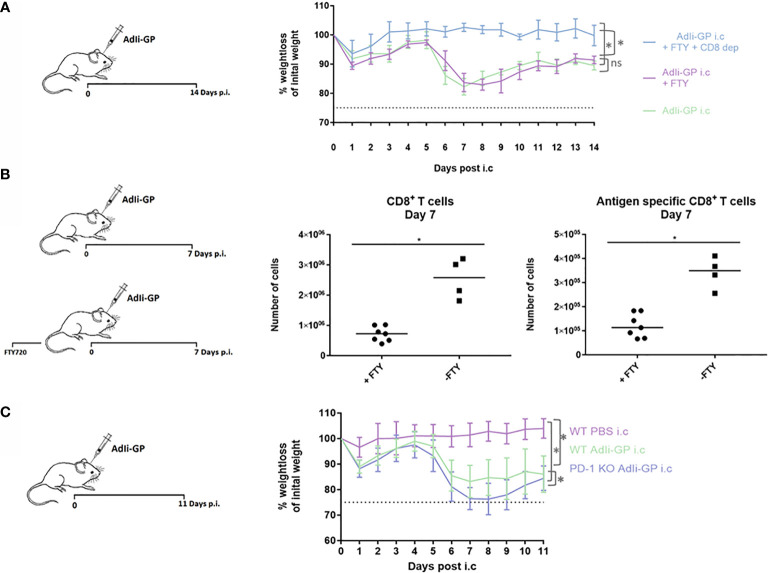
PD-1 dampens the T-cell mediated immunopathology caused by i.c. inoculation of AdIi-GP. Three groups of mice were inoculated i.c. with AdIi-GP. Two of the groups were administered FTY720 in their drinking water. One of the groups was additionally depleted from CD8^+^ T cells as described in M&M. **(A)** The percentage of initial bodyweight lost during the period from zero - 11 days post i.c. is depicted for all groups. **(B)** C57BL6 mice were inoculated i.c. with AdIi-GP and half of the mice were administered FTY720 prior to inoculation. The number of CNS infiltrating CD8^+^ and antigen specific CD8^+^ T cells extracted day 7 post i.c. is shown. Each dot represents an individual. Bars represent median. **(C)** C57BL/6 – and PD-1 KO mice were inoculated i.c. with AdIi-GP. Additionally, a control group of C57BL/6 mice were i.c inoculated with PBS. Weights were monitored from day zero - 11 and the percentage of initial bodyweight lost is shown. Means and standard deviations for 4-30 animals per group pooled from 3 experiments. For the weight curves, AUC was calculated for all mice and summed for each group. A significant difference in the AUC between the groups is indicated by * if P<0.05.

As robust T-cell infiltration to the CNS is a key aspect of our model-system, we speculated whether this could result in T-cell mediated neuropathology. Therefore, three groups of mice were inoculated i.c. with AdIi-GP. Two days prior to i.c. inoculation two of the groups were administered FTY720 in their drinking water. One group of mice was, in addition to the FTY720 treatment, depleted of CD8^+^ T cells by injections with CD8^+^ specific antibodies. Antibody injections were performed one day before i.c. inoculation and again three days post i.c. No signs of immunopathology were observed in the group, in which the mice were both depleted of CD8^+^ T cells and blocked for CD8^+^ T-cell recruitment to the CNS (**Figure 6A**). However, both groups of mice without any CD8^+^ T-cell depletion showed a significant decrease in bodyweight compared to mice depleted of CD8^+^ T cells (**Figure 6A**). On its own, the treatment with FTY720 did not cause a complete block in CD8^+^ T cell migration to the CNS, however, the treatment did reduce the early recruitment of CD8^+^ T cells by a factor of three to four fold (**Figure 6B**). This indicates that although the observed clinical disease is CD8^+^ T-cell dependent, there is no direct correlation between CD8^+^ T-cell numbers inside the CNS and the severity of the observed disease.

To analyze whether PD-1 expression modifies the severity of clinical disease following AdIi-GP i.c. inoculation, WT and PD-1 KO mice were inoculated i.c. with AdIi-GP. In addition, a group of WT mice inoculated with PBS was included as a control for the pathology induced by the inoculation trauma. Body weights of all mice were measured during a period from zero to 11 days p.i. In accordance with our previous findings of local antigen expression as an essential driver of CNS T-cell infiltration (37), no signs of clinical disease was observed in PBS inoculated WT mice (**Figure 6C**). In contrast, both groups of AdIi-GP inoculated mice showed a significant weight loss compared to the PBS control group (**Figure 6C**). Most interestingly, despite the fact that both groups of AdIi-GP inoculated mice show signs of recovery by the end of the observation period, the group of AdIi-GP vaccinated PD-1 KO mice lost significantly more body weight during the observation period, compared to WT AdIi-GP inoculated mice. This is clearly seen, if area under the curve (AUC) is calculated for both groups (**Figure 6C**). Based on these clinical data, we conclude that PD-1 expression on CD8^+^ T cells helps dampening the severity of the acute immunopathology caused by AdIi-GP induced local antigen expression within the CNS.

### PD-1 Expression Significantly Impact Both Numbers of Initially Recruited CD8^+^ T Cells as Well as Memory Cell Differentiation

**Figure 7:**
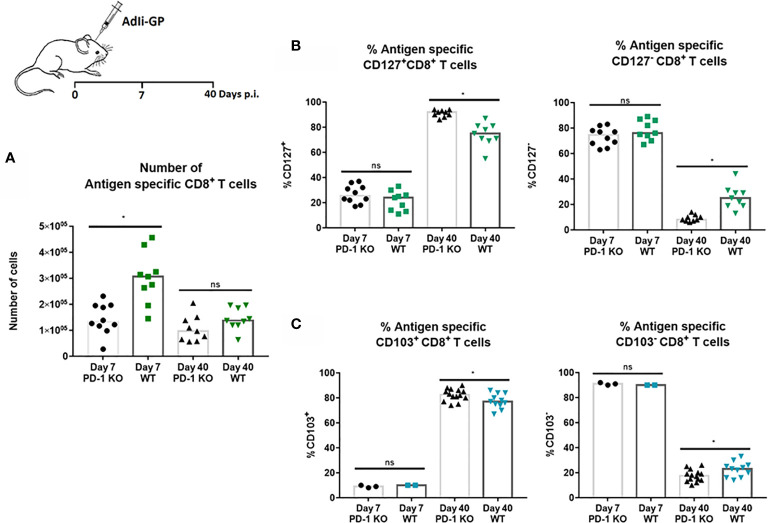
PD-1 expression affects numbers of CD8^+^ T cells initially recruited to CNS, and impacts CD8^+^Trm cell differentiation. WT C57BL/6 - and PD-1 mice were inoculated i.c. with AdIi-GP. **(A)** Seven and 40 days post i.c. the infiltrating antigen specific (tetramer^+^) CD8^+^ T cells were enumerated for both groups and compared. **(B)** The percentage of CD127 positive or CD127 negative antigen specific CD8^+^ T cells were compared for WT and PD-KO mice at both time points. **(C)** The percentage of antigen specific CD8^+^ T cells positive or negative for CD103 expression were analyzed and compared for WT and PD-1 KO mice. Each dot represents an individual, results are pooled from 2 experiments. Columns represent median. P>0.05 illustrated by *.

The above findings led us to speculate whether PD-1 expression had any direct effects on the CD8^+^ T-cell recruitment to the CNS. For that reason, numbers of CNS infiltrating antigen specific CD8^+^ T cells was analyzed 7 and 40 days post i.c inoculation in WT and PD-1 KO mice inoculated with 2x10^7^ pfu AdIi-GP. Interestingly, although the pathology was found to be more severe in PD-1 KO mice, significantly fewer antigen specific CD8^+^ T cells were recovered from the CNS of these mice during the effector phase, compared to WT mice **(** **Figure 7A**). In the memory phase analyzed at day 40 p.i., a significant difference in numbers of local antigen specific CD8^+^ T cells was no longer observed **(** **Figure 7A**).

Recent studies have pointed to a possible role of PD-1 in Trm differentiation (27, 36). For that reason, we decided also to analyze whether PD-1 expression affected the differentiation of recruited CD8^+^ T cell into canonical Trm cells. More specifically, we investigated whether PD-1 expression affected the expression of some of the cardinal markers for Trms, namely the expression of CD127 and CD103 (27, 52). As previously, WT and PD-1 KO mice were inoculated i.c with AdIi-GP and brains were harvested day 7 and 40 p.i. and antigen specific CD8^+^ T cells were analyzed for the expression of CD127 and CD103. At the early time-point, no difference was observed in neither the number nor percentage of CD8^+^ T cells expressing CD127 **(** **Figure 7B**). However, when comparing PD-1 KO and WT mice in the memory phase (day 40 p.i.), the picture was different. Thus, while absolute numbers of subtypes of CD8^+^ T cells were mostly of similar magnitude (see **Supplementary Figure 3**), the distribution of cells within the defined subsets was different: the percentage of cells positive for CD127 was significantly increased in PD-1 KO mice compared to WT mice **(** **Figure 7B**), as was the percentage of CD103 positive CD8^+^ T cells **(** **Figure 7C**). Therefore, unlike published data, these data indicate that PD-1 expression impairs the full development of canonical Trms in the CNS.

### Analysis of Transcriptional Factors Eomes and T-Bet in CD8^+^ Trm Cells Reveals No Signs of Exhaustion

**Figure 8:**
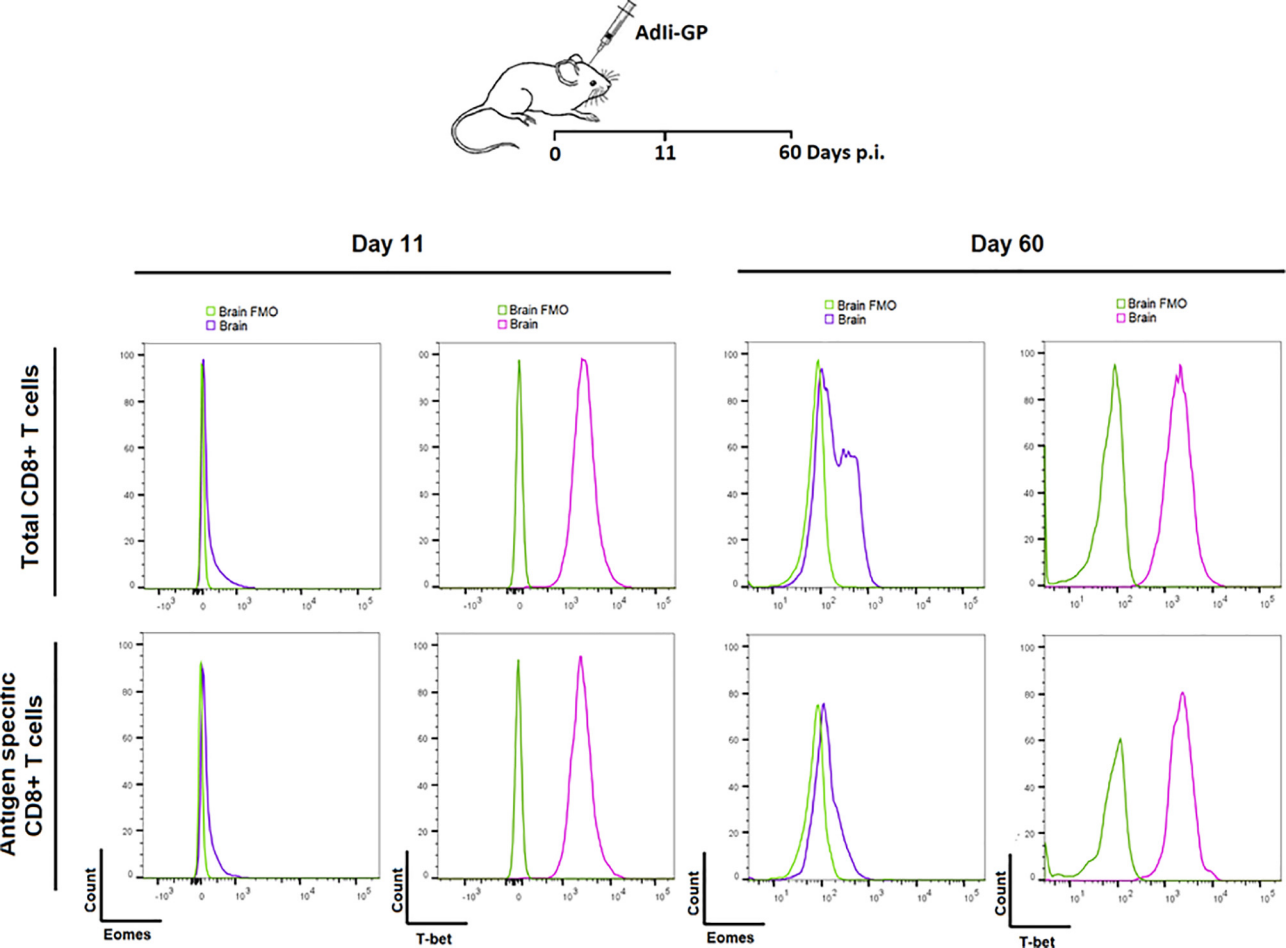
Expression of transcription factors Eomes and T-bet in the PD-1 positive CD8^+^ T cells during the acute and memory stage. Upon i.c. inoculation with AdIi-GP brains were extracted day 11 - and 60 post i.c. Intracellular expression of the transcription factors Eomes and T-bet were analyzed for both total CD8^+^ - and antigen specific (tetramer^+^) CD8^+^ T cells. The expressions were shown in representative histograms of 5 mice from each time point. FMOs were included to indicate background. Results are representative of 3 experiments.

Based on the above observations, we went on to investigate whether PD-1 expression had any impact on the functionallity of the induced CD8^+^ T cell population in the long-term. In particular we wanted to investigate whether the CNS induced CD8^+^ T cell population showed signs of exhaustion upon PD-1 expression. As a first thing we analyzed the transcriptional profile of the local CD8^+^ T cells. The relative levels of expression of the transcription factors T-bet and Eomes is an important indicator of the differentiation stage of memory CD8^+^ T cells (19, 53, 54). For that reason, the expression level of these specific transcriptionfactors were analyzed for the CNS CD8^+^ memory T-cell population induced upon AdIi-GP i.c inoculation. C57BL/6 mice were inoculated i.c with AdIi-GP and 11 and 60 days p.i., brains were extracted and CD8^+^ T cells were analyzed for the intracellular expression of T-bet and Eomes. The expression of Eomes, which is typically highly expressed in exhausted cells, was marginally elevated during both effector - and memory phase of the immune response (day 11 and 60, respectively) (**Figure 8**). In contrast, T-bet expression, which is expressed at intermediate levels by by Trm cells, was found to be expressed at both time-points (**Figure 8**). Notably, analysis of co-expression of these transcription factors did (data not shown) not reveal the dichotomy of memory CD8^+^ T cell subset typical of an exhausted population (19), instead the transcriptional profile of the AdIi-GP induced CD8^+^ T cell population within the CNS matches the transcriptional profile described for Trms in several peripheral organs (55).

### CNS CD8^+^ Memory T Cells Are Fully Functional

**Figure 9:**
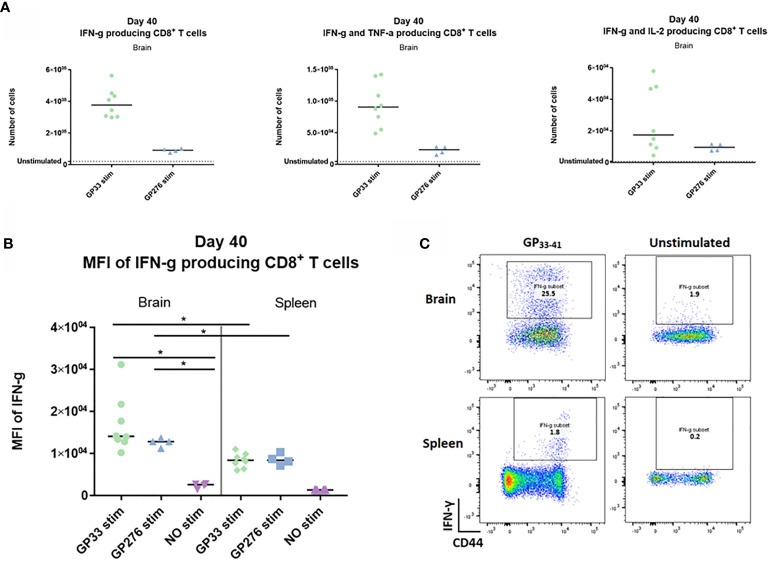
*Ex vivo* functional capacity of the PD-1 positive CD8^+^ T cell in the memory stage. C57BL/6 mice were inoculated with AdIi-GP i.c and at day 40 post i.c. numbers of CNS derived CD8^+^ T cells producing IFN-γ, IFN-γ and TNF-α – or IL-2 in response to *in vitro* GP_33-41_ or GP_276-281_ stimulation were enumerated as shown. **(A)** Unstimulated samples were used to set the background. **(B)** The mean fluorescence intensity (MFI) of the IFN-γ response was calculated for CD8^+^ T cells extracted from both brain and spleen. Unstimulated samples were included as control. **(C)** representative plots of cytokine producing CD8^+^ T cells in presence or absence of peptide stimulation. Results are representative of 2–3 experiments involving 4-5 mice/group.

Next, we wanted to investigate whether the memory CD8^+^ T cells generated upon i.c. inoculation showed any evidence of being functionally exhausted. To this end, we analyzed the cytokine-producing abilities of identical memory CD8^+^ T cell populations recovered from either spleen or CNS. Forty days post i.c. inoculation with AdIi-GP, brain and spleen derived CD8^+^ T cells were harvested and restimulated *in vitro* with peptides representing two of the major epitopes encoded in the adeoviral-construct, namely LCMV GP_33-41_ and GP_276-281_. A sample of both brain and spleen cells were left unstimulated to determine background expression. The number of CNS derived CD8^+^ T cells producing IFN-γ, IFN-γ and TNF-α – or IL-2 were enumerated as shown in **Figure 8A**. Although the response varied in accordance with the immunodominance hierarchy, the production of all three cytokines was significantly increased compared to the unstimulated sample (**Figure 9A**). In particular, the production of IFN-γ and TNF-α was markedly increased upon *in vitro* recognition of the cognate antigen (**Figure 9A**). To compare per cell capacity for cytokine production, mean fluorescence intensity (MFI) of the IFN-γ signal was determined for antigen specific cells harvested not only from the CNS, but also for cells with the same antigen specificity in the spleen of the same mice. Interestingly, when we compared the MFI values for the PD-1^+^ neuro-inflammatory CD8^+^ T cells with their PD-1^-^ counterparts in the spleen, a significantly higher average MFI value was noted for the CNS derived population (**Figures 9B, C**). Consequently, these data strongly indicate, that the CNS localized memory CD8^+^ T-cell population intrinsically is not functionally impaired despite their PD-1^+^ phenotype.

**Figure 10:**
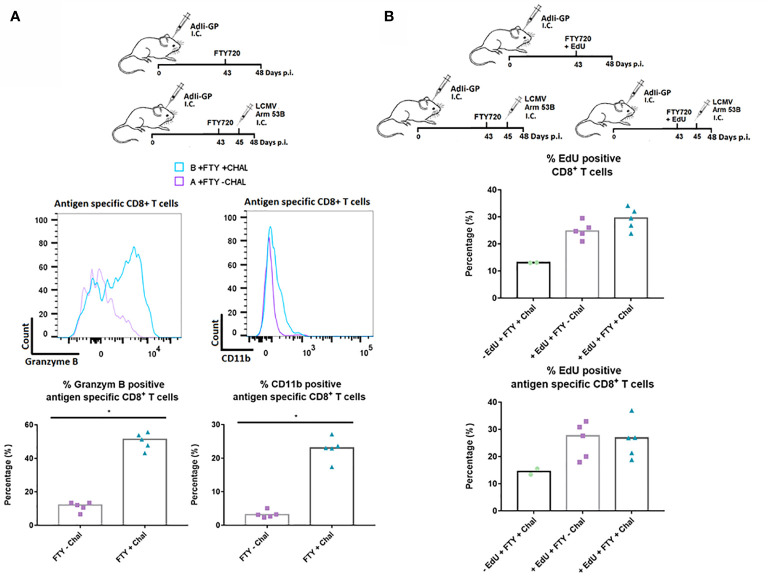
*In situ* functional capacity of the PD-1 positive CD8^+^ T cell in the memory stage. C57BL/6 mice were inoculated with AdIi-GP i.c and FTY720 was administered to all mice day 43 post inoculation. *In vivo* challenge was performed for half of the mice by i.c. infection with LCMV Armstrong two days later (day 45 p.i.). Antigen specific (tetramer^+^) CD8^+^ T cells were analyzed. **(A)** Representative histograms of 5 mice showing the content of Granzyme B and the expression of CD11b for challenged and unchallenged mice, respectively. These data are also graphically presented as the percentage of antigen specific (tetramer^+^) CD8^+^ T cells positive for Granzyme B and CD11b. **(B)** Three groups of C57BL/6 mice were administered FTY720 43 days post i.c. inoculation with AdIi-GP. Group A and B were administered EdU and group C was left as a negative control for EdU incorporation. Group B and C were challenged i.c. with LCMV Armstrong at day 45 p.i. The percentage of CD8^+^ and antigen specific CD8^+^ T cells positive for EdU are shown. Each dot represents an individual. Columns represent median. P < 0.05 illustrated by *.

Finally, to study the responsiveness of the CNS Trms in situ, we sat up an additional experiment where we evaluated the capacity of the cells to become reactivated during local antigen challenge. To assure that the measured response reflected the response of the local cells, mice were administered FTY720 in their drinking water 43 days after induction of a Trm population using AdIi-GP i.c. inoculation. Two days later, part of the mice was challenged i.c. with an otherwise lethal dose of LCMV Armstrong. At three days post-challenge brains were harvested from all animals and the expression of granzyme B as well as CD11b was used to gauge the *in vivo* response of the antigen specific CD8^+^ T cells. A significantly increased expression of granzyme B was induced following challenge with LCMV Armstrong. In a previous study, we have clearly correlated the expression of CD11b to the activation state of the CD8^+^ T cells, and matching the increase in granzyme B expression, we also observed an increased CD11b expression following challenge with LCMV (**Figure 10A**) (56). We also looked for increased incorporation of EdU, however, no increase was observed for the challenged mice (**Figure 10B**). This probably indicates that the re-infection never reached a level sufficient to drive a proliferative response. Collectively, the above data strongly indicate that the antigen specific CD8^+^ T cells located within the CNS are functionally capable of respounding upon antigen stimulation, not only *in vitro*, but also *in vivo*.

## Discussion

Trms present inside the CNS constitute a population of primed sentinel cells, which provides the CNS with long-term protective immunity against recurrent or reactivated brain-infections. However, to limit immunopathology, the efficient effector mechanisms held by this memory subset needs to be tightly controlled. Trms and their effector mechanisms have been studied in various organs (1, 4, 57–59). Despite that, our general knowledge about the mechanisms involved in restricting these functions within a non-regenerative tissue like the brain, is limited.

In this present study, we provide evidence that point to an immune modulating role of PD-1 expression by brain infiltrating CD8^+^ T cells. This analysis was conducted utilizing a recently established *in vivo* model for generating Trms within the CNS (37). By intracerebral inoculation with a non-replicating adenoviral vector we were able to induce long-lasting CD8^+^ T cell memory within the CNS. In accordance with studies of brain Trms cells carried out in the context of chronic viral infections, we found sustained PD-1 expression by the CNS infiltrating CD8^+^ T cells (28, 33). An impact of PD-1 on effector capacity can, however, only be relevant if the involved cells encounter their relevant ligand. The high number of IFN-γ producing effector CD8^+^ T cells recruited to the CNS would likely stimulate PD-L1 upregulation, as IFN-γ has been found to be a potent inducer of PD-L1 expression (22, 29). However, expression of PD-L1 by the different cell subsets in the inflamed CNS, seem to vary dependent on the source and duration of infection (24, 29, 30). Our findings of PD-L1 upregulation on various infiltrating and resident CNS cells are in agreement with the observations found for PD-L1 expression on CNS cells during mouse polyomavirus (MuPyV) infection (22). Similar to the situation predicted in our model system, MuPyV infection results in repetitive exposure of low-level of antigen. This was shown to result in PD-L1 expression on infiltrating monocytes, microglia and astrocytes, whereas PD-L1 expression on oligodendrocytes was not detected (22). Accordingly, we found markedly elevated PD-L1 expression by the recruited innate immune cells compared to the expression of PD-L1 on the resident CNS cells. The observed decrease in PD-L1 expression with time likely reflects a decrease in the stimulation of the recruited T cells as the infection subsides (indicated by the decreasing level of CD43 expression (**Figure 3C**), see further below), resulting in reduced levels of IFN-γ. It should be noted, however, that although the expression of PD-L1 decreased with time, distinct expression was maintained by some cell types at least until day 60 post i.c. permitting some PD-1:PD-L1 interaction also long-term.

In accordance with some previous studies of Trm cells present inside the CNS, we found the recruited CD8^+^ T cells to express additional inhibitory receptors linked to T-cell exhaustion (28, 60). As with PD-L1, the expression of Tim-3 and Lag-3 decreased with time. This was found to correlate with decreased signs of recent CD8^+^ T-cell activation and proliferation, as indicated by a decrease in the percentage of CD8^+^ T cells expressing CD43 (48). Although our results do not directly evaluate the link between antigenic load and receptor expression, the decrease in CD43 likely reflects a decrease in antigen expression (47), indicating a correlation between local antigen and sustained expression of inhibitory markers expressed by the CD8^+^ T cells. We, and others have previous shown local antigen expression to be pivotal in upregulation of CD103 by brain localized Trms (4, 37, 45). This is, however, not the case in several other tissues (52, 61, 62), which led us to wonder whether antigen or a local inflammatory environment would be key inducer of PD-1 expression by memory CD8^+^ T cells localized in the brain. Although we did find clear PD-1 expression in the context of bystander inflammation, our data points to a clear relation between local antigen encounter and level of PD-1 expression. The PD-1 expression was more pronounced in the presence of local antigen than as a result of bystander inflammation, indicating that although factors in the CNS environment seem to drive PD-1 expression, TCR signaling within the CNS critically increase PD-1 expression by the local CD8^+^ T cells. In accordance with our findings regarding prolonged PD-1 expression, a recent study of Shwetank et al. showed that despite equivalent viral loads in bran and spleen, the *Pdcd1* promotor remained more extensively demethylated in brain Trms, suggesting that the brain milieu somehow favors PD-1 expression (33). Interestingly, similar to the PD-1 receptor, CD39 expression did not decrease over time. Although several studies have reported CD39 as a marker related to T-cell exhaustion and death, CD39 has also been found to possess nuclease activity and thereby the ability to generate extracellular adenosine through ATP hydrolysis (63, 64). ATP concentrations are high in inflamed tissue, like the CNS in our model settings, and CD39 could likely convert this high concentration of ATP into adenosine. Adenosine signaling have been found to inhibit the TCR mediated activation of the Akt-pathway, which otherwise contribute to T cell differentiation (65–67). Thus, CD39 likely initiates Akt-inhibition in the T cell by generating adenosine and *via* Akt-inhibition contribute to regulating the fate of the T cell by controlling the differentiation into SLEC or MPEC (68). Accordingly, this Akt-inhibition have been associated with enhanced formation of long lived memory CD8^+^ T cells and enhanced T cell persistence (65, 66). Furthermore, to underline the important role of CD39 expression as an immune regulatory receptor, T cell activation in the presence of an agonist for the adenosine receptor, have been reported to generate Tregs with stronger immune regulatory potential (69). Conversely, deficiency in the receptor responsive to adenosine was reported to result in reduced CD127 (70) which are in line with findings showing that adenosine signaling are necessary for CD127 expression and survival of long-lived memory CD8^+^ T cell (70, 71). When we, in a previous study analyzed the expression of CD127, we found that the portion of CD8^+^ T cells showing moderate expression of CD127 was higher in the CNS than in the spleen (37). Both the expression of CD127 and PD-1 by brain resident Trms have been linked to prolonged or chronic antigen expression (27). However, when we in this present study, correlated CD127 expression with PD-1, we found a reciprocal expression pattern of PD-1 and CD127. This let us to speculate whether PD-1 expression could influence memory differentiation. Conflicting results have been found with regard to the correlation between PD-1 expression and the canonical Trm markers CD127 and CD103 (22, 27). Reduced expression of CD127 have been found in both PD-1 and PD-L1 KO mice, and have been linked to prolonged effector like T-cell responses in the CNS of mice chronically infected with murine cytomegalovirus (MCMV) (27). In contrast, we found a significant decrease in the percentage of of antigen specific memory CD8 T cells expressing either CD127 or CD103 in WT mice compared to those from matched PD-1 KO mice. Rather than indicating a prolonged effector T-cell response, we believe these findings suggest that PD-1 expression could influence not only T-cell effector capacity, but also impact the differentiation of the memory CD8^+^ T cell population within the CNS. Together with the prolonged expression of CD39, we believe these markers could act as counterparts for balancing the generation of long-lived memory CD8^+^ T cells within the CNS.

When investigating the clinical consequences related to intracranial inoculation of AdIi-GP, we observed that PD-1 expression by the CD8^+^ T cells helps dampening the severity of the acute immunopathology generated upon AdIi-GP induced, local antigen expression within the CNS. A lower number of antigen specific CD8^+^ T cells were recruited to the CNS of PD-1 KO mice, yet these mice experienced a slightly more severe clinical course than matched WT mice. However, although the observed immunopathology was clearly associated with CD8^+^ T-cell recruitment, we found no direct correlation between CD8^+^ T-cell numbers and clinical severity. This observation was also made comparing the clinical course for untreated WT mice versus WT mice treated with FTY720. Based on these data, it is tempting to conclude that although recruited in higher numbers, antigen specific WT CD8^+^ T cells cause less disease than cells lacking PD-1 expression, indicating that the activation state of the infiltrating CD8^+^ T cells, rather than their absolute numbers is critical in mediating neuropathology.

The most striking finding obtained during this study was obtained when we analyzed the functionality of the PD-1 positive CD8^+^ T cells in CNS. In line with several studies showing that CD8^+^ T cells with a restrained phenotype retain the ability to contribute to viral control (13, 19, 46, 72), we found, that despite the exhausted phenotype of CNS resident CD8^+^ T cells, these cells were nevertheless able to produce a potent cytokine response upon restimulation ex vivo. Indeed, the potential for cytokine expression measured in terms of the per cell expression of intracellular cytokine storage was significantly higher for PD-1^+^ antigen specific CD8^+^ T cells from CNS than for PD-1^-^ splenic memory cells of the same specificity. The additional results obtained when analyzing the transcriptional profile of the CD8^+^ T cells maintained in the CNS, also points to a functional memory subset, as we found a T-bet^int^ Eomes^low^ phenotype. This does not match what others have found for populations of exhausted CD8^+^ T cells (19), but rather fits the transcriptional profile found for Trms in several peripheral organs (55, 73). Additionally, our experiments assessing the capacity of the CD8^+^ T cells to respond to direct intracerebral challenge with LCMV Armstrong revealed that re-infection drove a distinct upregulation of CD11b and led to increased intracellular expression of granzyme B in the antigen specific CD8^+^ T cells. Although we did not observe increased local proliferation as a response to rechallenge, we did find that the bona fide CNS CD8^+^ Trm cells retained the capacity to undergo local homeostatic proliferation to maintain the population despite being PD-1 positive.

Taken together this study contributes with insight into the mechanisms involved in maintaining and regulating immune responses within the CNS. Our results revealed a functionally capable memory CD8^+^ T cells population inside the CNS, despite expression of PD-1. This population is maintained, at least in part, through local homeostatic proliferation. That is not to say, that PD-1 does not exert any relevant immune-regulatory role inside CNS. First of all, during the acute phase of the response, when there is active inflammation, the severity of this is modulated by PD-1:PD-L1 interaction. Second, expression of PD-1 significantly modifies the relative phenotypic composition of the memory CD8^+^ T cells found following establishment of a local Trm population. Based on these results we suggest that rather than serving as a marker of epigenetically modified cells, PD-1 on Trms in the CNS represent a fast switch to rapidly modulate T cell induced inflammation in a highly vulnerable organ. Hopefully a better understanding of immune regulation inside CNS will be of benefit in the search for new targets of treatment for diseases like acute disseminated encephalomyelitis (ADEM) and MS.

## Data Availability Statement

The raw data supporting the conclusions of this article will be made available by the authors, without undue reservation.

## Ethics Statement

Experiments were conducted in accordance with national Danish guidelines (Amendment # 1306 of November 23, 2007) regarding animal experiments as approved by the Danish Animal Experiments Inspectorate, Ministry of Justice, permission number 2015-15-0201-00623.

## Author Contributions

AS, LN, JC, and AT designed the study. AS and LN performed the experiments and analyzed the data. AS, LN, JC, and AT interpreted the data. AS and AT drafted the manuscript. All authors contributed to the article and approved the submitted version.

## Funding

This work was supported by Scleroseforeningen (A33255 and A37864), Warwara Larsen’s Fund, the Danish Medical Research Council (7016-00234) and Faculty of Health and Medical Sciences, University of Copenhagen.

## Conflict of Interest

The authors declare that the research was conducted in the absence of any commercial or financial relationships that could be construed as a potential conflict of interest.
