# Supplementary figures and images for: Functionally Competent, PD-1+ CD8+ Trm Cells Populate the Brain Following Local Antigen Encounter
Source: Front Immunol. 2021 Feb 2;11:595707. doi: 10.3389/fimmu.2020.595707 (PMC7884456; doi:10.3389/fimmu.2020.595707)

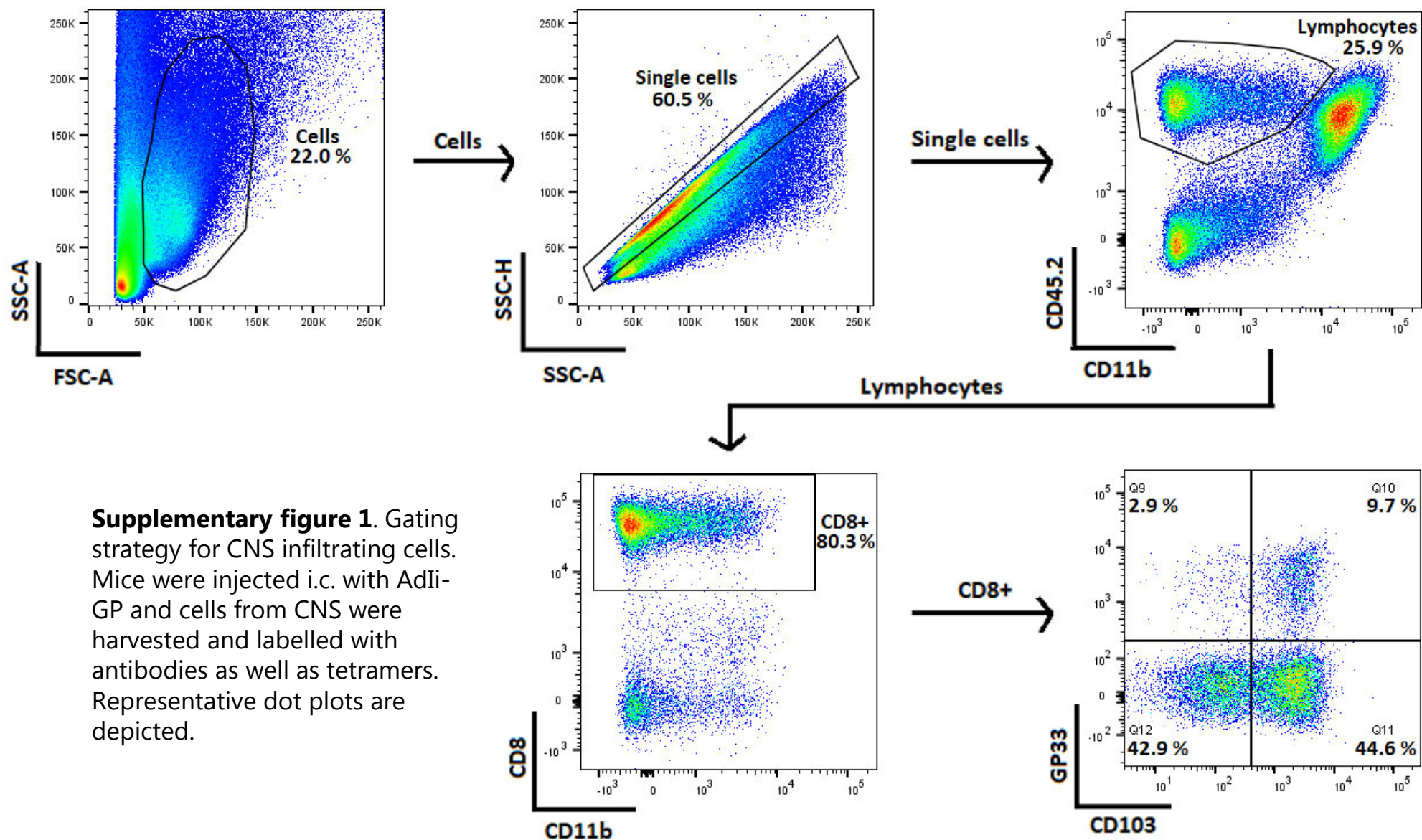

Supplement: Supplementary file 1 [file DataSheet_1.pdf]
