# Supplementary material for: Functionally Competent, PD-1+ CD8+ Trm Cells Populate the Brain Following Local Antigen Encounter
Source: Front Immunol. 2021 Feb 2;11:595707. doi: 10.3389/fimmu.2020.595707 (PMC7884456; doi:10.3389/fimmu.2020.595707)
Supplement: Supplementary file 2 [file DataSheet_2.pdf]

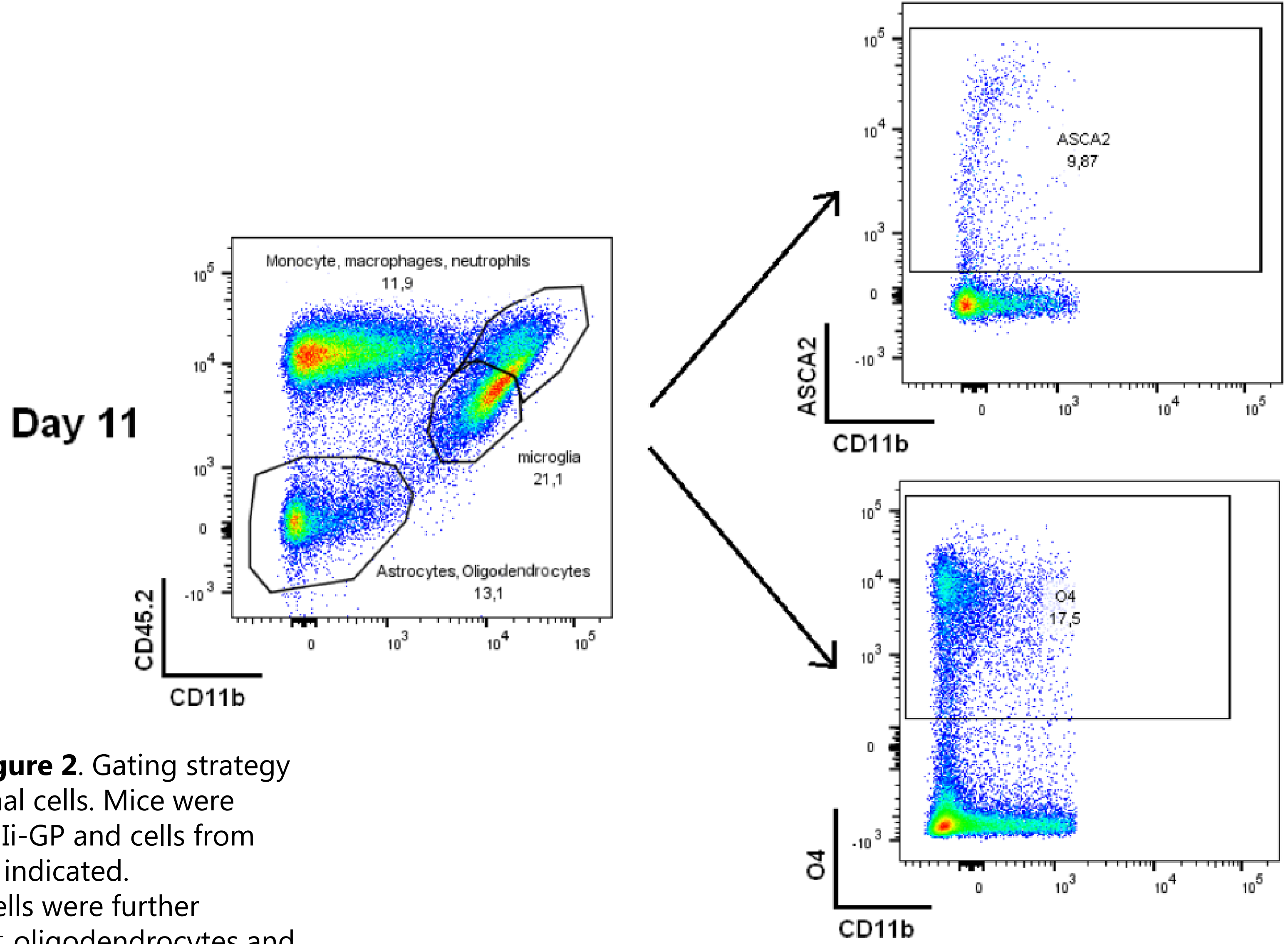

Supplementary figure 2. Gating strategy for CNS parenchymal cells. Mice were injected i.c. with AdIi-GP and cells from CNS were gated as indicated. CD45^{low}CD11b^{low} cells were further subdivided into 04^{+} oligodendrocytes and ASCA2^{+} astrocytes. Representative dot plots from a mouse inoculated 11 days earlier are depicted.
