# Supplementary material for: Functionally Competent, PD-1+ CD8+ Trm Cells Populate the Brain Following Local Antigen Encounter
Source: Front Immunol. 2021 Feb 2;11:595707. doi: 10.3389/fimmu.2020.595707 (PMC7884456; doi:10.3389/fimmu.2020.595707)
Supplement: Supplementary file 3 [file DataSheet_3.pdf]

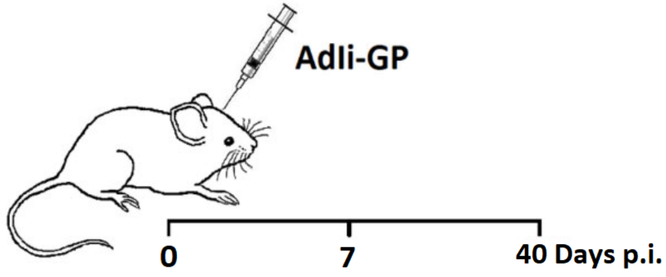

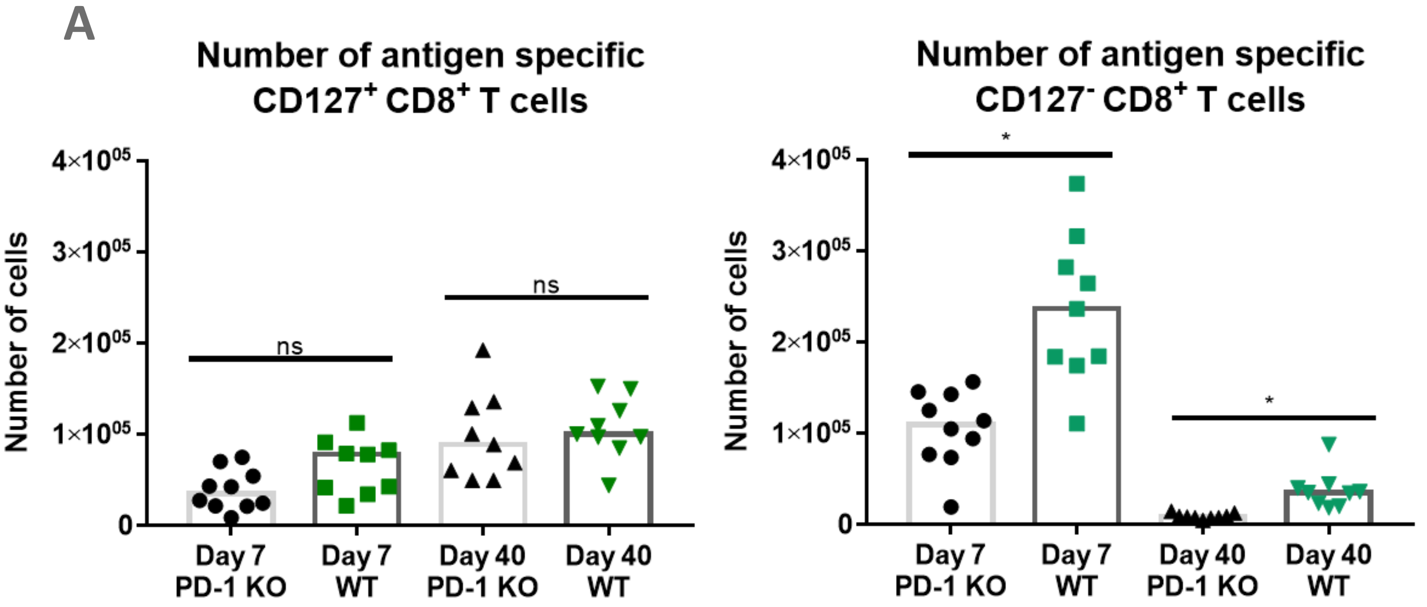

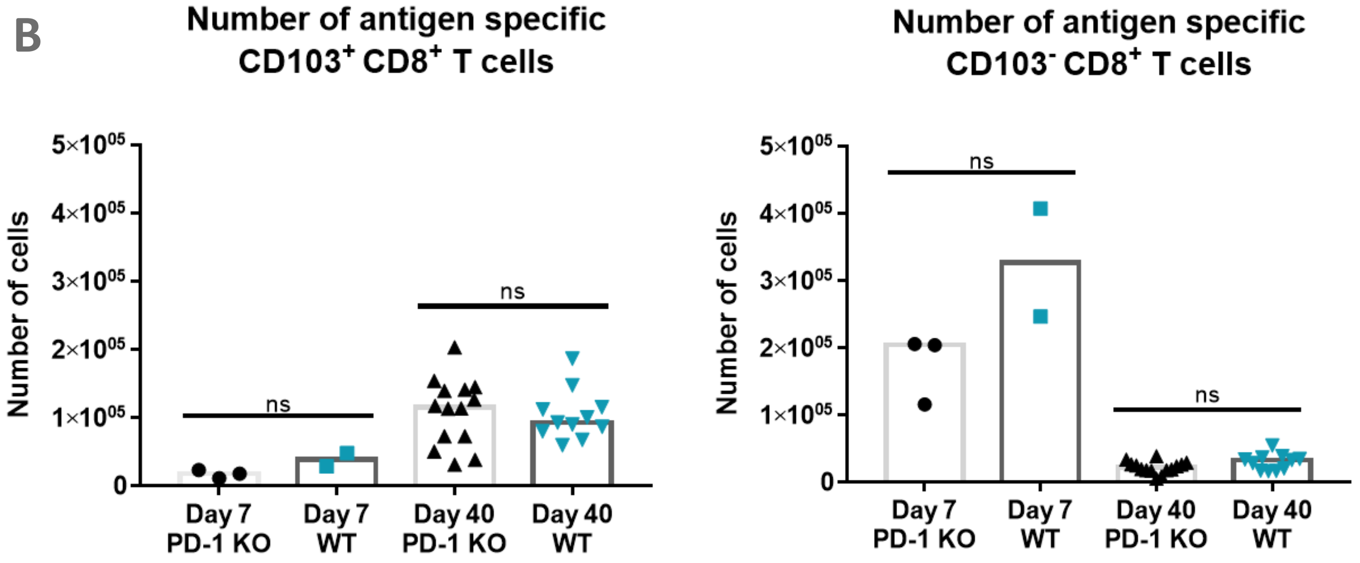

Supplementary figure 3. WT C57BL/6 - and PD-1 mice were inoculated i.c. with AdIi-GP and seven and 40 days post i.c. the infiltrating antigen specific (tetramer$^+$) CD8$^+$ T cells for both groups were analyzed. A) Numbers of CD127 positive or CD127 negative antigen specific CD8$^+$ T cells in WT and PD-KO mice at both time points. C) Numbers of antigen specific CD8$^+$ T cells positive or negative for CD103 expression in WT and PD-1 KO mice. Each dot represents an individual, results are pooled from 2 experiments. Columns represent median. P>0.05 illustrated by *
